# Sweet tooth: Elephants detect fruit sugar levels based on scent alone

**DOI:** 10.1002/ece3.6777

**Published:** 2020-09-11

**Authors:** Omer Nevo, Melissa H. Schmitt, Manfred Ayasse, Kim Valenta

**Affiliations:** ^1^ Institute of Evolutionary Ecology and Conservation Genomics Ulm University Ulm Germany; ^2^ German Centre for Integrative Biodiversity Research (iDiv) Halle‐Jena‐Leipzig Leipzig Germany; ^3^ Institute of Biodiversity Friedrich Schiller University Jena Jena Germany; ^4^ Department of Ecology, Evolution, and Marine Biology University of California Santa Barbara Santa Barbara CA USA; ^5^ South African Environmental Observation Network Ndlovu Node Phalaborwa South Africa; ^6^ Department of Anthropology University of Florida Gainesville FL USA

**Keywords:** animal–plant interactions, chemical communication, frugivory, honest signaling, olfaction, seed dispersal

## Abstract

The ability to assess food quality is crucial to all organisms. Fleshy fruits are a major source of nutrients to various animals, and unlike most food sources, have evolved to be attractive and to be consumed by animals to promote seed dispersal. It has recently been established that fruit scent—the bouquet of volatile chemicals emitted by ripe fruit—is an evolved communication system between plants and animals. Further, it has been argued that chemicals that are synthesized from sugar and its products may be an honest signal for sugar content and fruit quality. Elephants are important seed dispersers for numerous species and possess an olfactory system that is likely to outperform most other animals. We tested the hypothesis that fruit scent signifies sugar content and that elephants are capable of assessing fruit sugar levels based on scent alone. Using a paired‐choice test of marula fruits (*Sclerocarya birrea*) by semitame African elephants, we show that elephants are capable of identifying more sugar‐rich fruits based on scent alone and that this is likely based on two chemical compounds: ethanol and ethyl acetate, both downstream products of sugar fermentation. These results shed light on the mechanisms driving elephant feeding ecology, plant signaling, and the coevolutionary process between angiosperms and animal seed dispersers.

## Graphical abstract

Elephants possess an excellent sense of smell that may fulfill many functions in their ecology and social lives, including feeding on fruit. Fruits have evolved to communicate with animal seed dispersers via chemical signals. We show that elephants are capable of detecting fruit sugar levels based on scent alone and identify chemical compounds that are associated with sugar content which may allow elephants to do so.

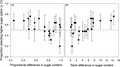

## 1 INTRODUCTION

The nutritional value of foods can vary tremendously, as both plants and animals have evolved a variety of defenses against predation (Farmer, 2014), are infected by organisms that render their host unpalatable (e.g., mold) (Janzen, 1977), or are selected to be attractive to mutualists while remaining toxic or unpalatable to others (Cipollini & Levey, 1997a; Valenta & Nevo, 2020). Fleshy fruits are a major source of macro‐ and micronutrients for many animals, but also vary tremendously in their value because they can contain secondary metabolites that range from harmful (e.g., tannins) (Chung, Wong, Wei, Huang, & Lin, 1998) to poisonous (e.g., cyanide) (Cipollini & Levey, 1997b). Thus, the ability of fruit‐eating animals to regularly detect and select fruits with the required nutrients, and avoid harmful and poisonous compounds, is critical to their survival and fitness.

Given that animals face countless feeding decisions each day, must meet basic nutritional requirements to function, and avoid overly harmful compounds, the ability to detect food quality should be under strong selection. Potential sources of information include gustatory (Baldwin, Dechmann, Thies, & Whitehead, 2020) and visual cues (Albrecht, Hagge, Schabo, Schaefer, & Farwig, 2018; Schaefer, Valido, & Jordano, 2014). Similarly, olfactory cues are used by animals to detect and identify ripe fruit (Hodgkison et al., 2007, 2013; Nevo et al., 2015; Nevo & Heymann, 2015; Nevo, Razafimandimby, Jeffrey, Schulz, & Ayasse, 2018). It has also been suggested that chemical compounds emitted by fruits may convey information on fruit quality. Primary metabolites such as methanol and ethanol are direct products of fruit maturation and were suggested to be positively associated with sugar and act as attractants (Sánchez et al. 2006; Dudley 2004). Building on these propositions, a recent study reported that across species, the presence and amount of various secondary metabolites in fruit scent are correlated with sugar levels, which indicates that fruit scent may be an honest signal of fruit quality (Nevo et al., 2019). In particular, this study emphasized the role of methyl and ethyl esters, two compound classes whose presence has been hypothesized to be biochemically linked to sugar content (Nevo & Ayasse, 2019; Nevo & Valenta, 2018). Both, like all aliphatic esters, are synthesized from an alcohol and acyl‐CoA (Beekwilder et al., 2004). The alcohol in methyl esters is methanol, which is a product of cell‐wall degradation and thus fruit maturation, while the alcohol in ethyl esters is ethanol, a product of sugar fermentation (Nevo & Ayasse, 2019). Thus, these results indicate that the relevant cue for animals may not be primary metabolites as suggested by Dudley (2004) and Sánchez et al. (2006), but rather secondary compounds synthesized from them. These results also provided the first empirical evidence for a biochemical link between chemical signals and rewards in fleshy fruits. As such, they provide further evidence for the hypothesis that fruit scent is an evolved signal to animal seed dispersal (Borges, Bessière, & Hossaert‐McKey, 2008; Hodgkison et al., 2013; Nevo, Heymann, Schulz, & Ayasse, 2016; Nevo et al., 2018; van der Pijl, 1982) because a direct link between signal and reward is considered the most likely substrate on which honest signals evolve (Schaefer & Ruxton, 2011). However, focusing on cross‐species patterns, these results remain only suggestive because (a) in order to be useful for frugivores, the relationship between signal and reward should also be held *within plant* species; and (b) there is to date no evidence that frugivores are capable of using fruit scent to identify sugar levels.

Elephants are notable among animals in their olfactory capabilities. Their olfactory system is equipped with about 2,000 different functional olfactory genes—the basis for acute olfactory discrimination capacities among vertebrates (Niimura, Matsui, & Touhara, 2014). This number is larger by a factor of ~2 relative to the next most olfaction‐oriented mammals, for example, dogs and rats, and by a factor of ~4 relative to humans and closely related primate taxa (Niimura et al., 2014; Niimura, Matsui, & Touhara, 2018). This translates into very high performance in olfactory tests. For example, Asian elephants are capable of discriminating between enantiomers (chemical compounds that are mirror images of each other and are thus functionally and chemically almost identical) at extremely high rates that are matched only by mice, and outperform primates, bees, and seals in detecting them (Rizvanovic, Amundin, & Laska, 2013). African Elephants use their sense of smell to find patches of feeding plants, but also to identify preferred species within a patch (McArthur, Finnerty, Schmitt, Shuttleworth, & Shrader, 2019; Schmitt, Shuttleworth, Ward, & Shrader, 2018), probably by perceiving which plant species contain more toxic or unpalatable secondary metabolites (Schmitt, Shuttleworth, Shrader, & Ward, 2020), or more nutrients. In controlled experiments, using scent alone, Asian elephants were able to locate food in an object‐choice task (Plotnik, Shaw, Brubaker, Tiller, & Clayton, 2014) and to differentiate between food quantities (Plotnik et al., 2019).

In addition to a large proportion of vegetative plant material, elephants also consume significant amounts of ripe fruits and thus provide seed dispersal services for a variety of plants (Campos‐Arceiz & Blake, 2011; Campos‐Arceiz et al., 2008; Cochrane, 2003). In particular, elephants disperse “megafanual fruits”—large and strongly protected fruits that have evolved to be dispersed by large frugivores (Guimarães, Galetti, & Jordano, 2008). Thus, as fruit‐eating animals with an exceptionally acute olfactory system, elephants are an ideal model system to test the hypothesis that fruit sugar levels can be inferred using scent alone.

Here, we test the hypothesis that fruit scent conveys honest information regarding sugar content through increased emission of aliphatic esters, and that fruit‐eating animals—in this case elephants—are capable of recognizing fruit sugar content based on those signals. We use African elephants and marula fruits (*Sclerocarya birrea*: Anacardiaceae) as a model system. We test the predictions that (a) elephants can identify and systematically choose fruits with higher sugar content based only on olfactory cues; and (b) sugar levels are predicted by the amount of aliphatic esters or ethanol emitted by fruits. We conducted choice experiments (Supplementary Video S1) between ripe marula fruits in which only olfactory cues were available and analyzed fruit scent chemistry and sugar content. We show that elephants choose fruits with higher sugar levels based on smell alone.

## 2 RESULTS

### 2.1 Behavioral experiment

**FIGURE 1:**
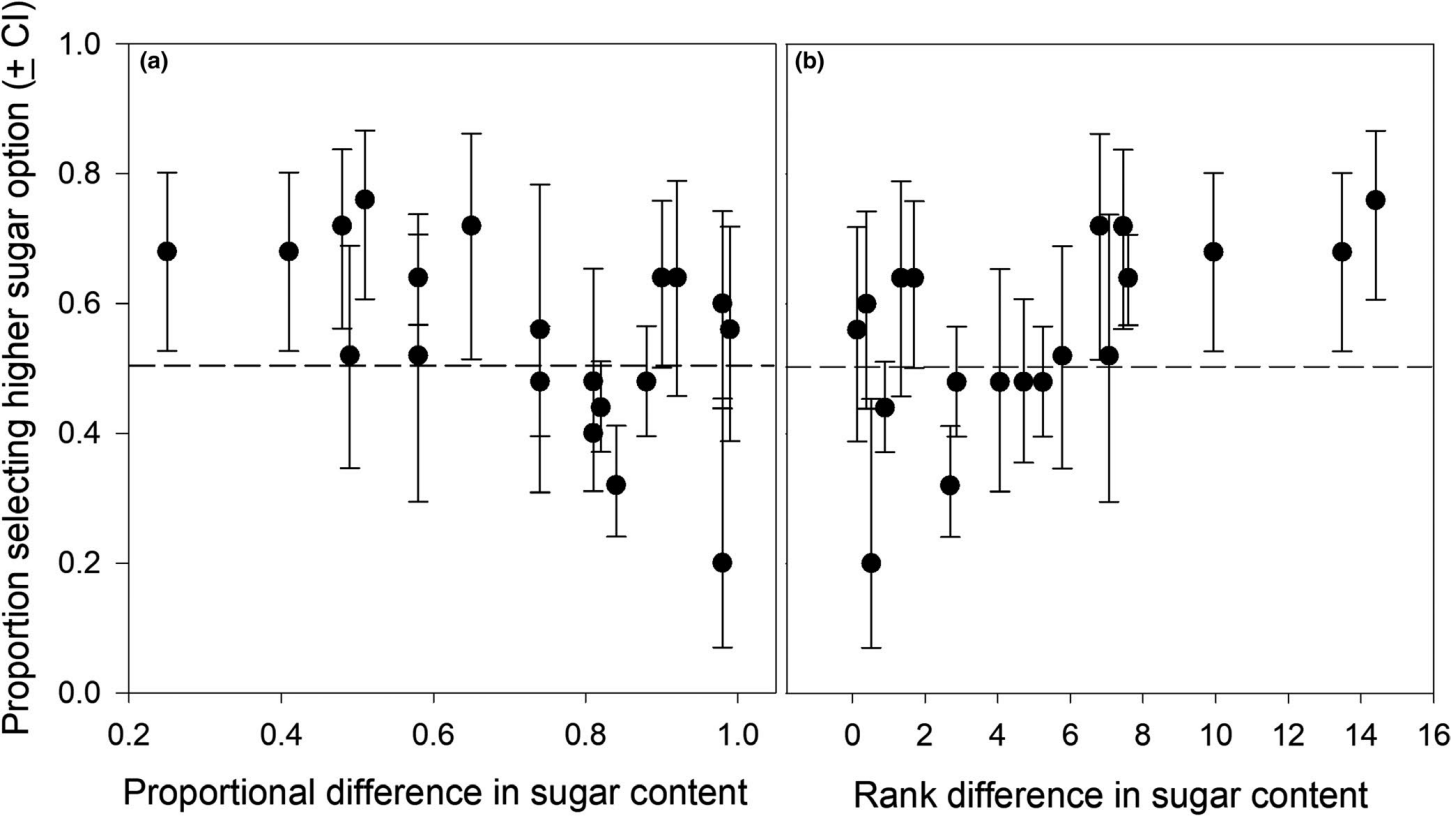
Proportion of elephants selecting the more sugar‐rich option as a function of (a) proportional difference in percent sugar content (ratio of % sugar between a pair of fruits), and (b) rank difference in percent sugar content between pairs of fruits. Marginal means (±95% confidence intervals) of the proportion of selection of a given option are plotted. Error bars overlapping the 0.5 expectation (i.e., random selection, dashed line) indicate no difference in preference. The two analyses demonstrate that differences in both relative (a) and absolute (b) amounts of sugar between fruits in a pair are associated with higher selectivity among the elephants

Elephants were able to choose fruits with higher sugar content in a blind, odor‐based choice experiment. This was driven by both the proportional difference (Figure 1a) and absolute difference (Figure 1b) in sugar content between the pair of fruits offered. First, a bigger difference in the proportion of sugar between two fruits in a pair was associated with increased selection of the higher sugar containing fruit (GEE: χ^2^ = 18.08, *p* < .001, Figure 1a). Similarly, a significant bias toward more sugar‐rich fruits was observed once the difference between the two options was greater than ~7% (GEE: χ^2^ = 38.8, *p* < .001, Figure 1b), indicating an increased ability to discriminate between the fruit, or alternatively stronger preference for the more sugary fruit.

### 2.2 Chemical association between scent and sugar

The scent profile of marula was strongly dominated by aliphatic esters, which constituted 64 ± 13% of the scent bouquet, of which by far the most dominant compound was ethyl acetate (30 ± 9%). The other dominant compound was ethanol (27 ± 11% of total VOC emissions) (Table S1).

**FIGURE 2:**
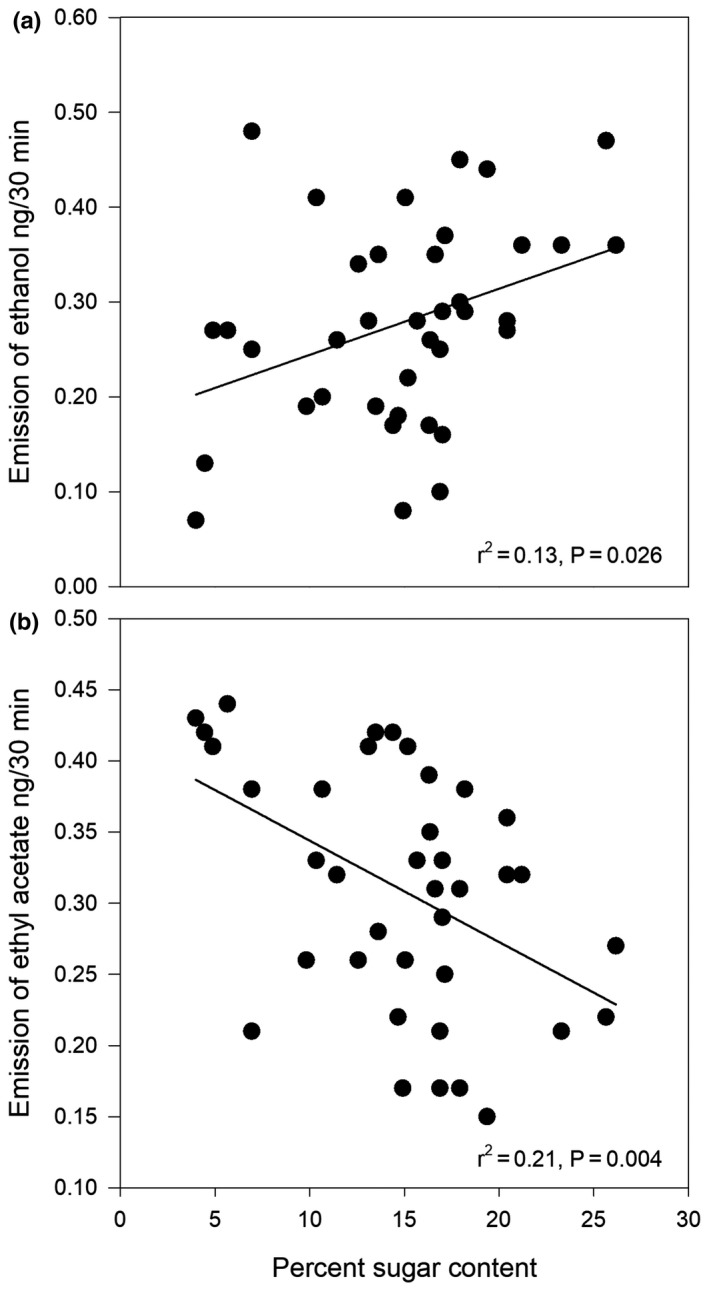
Relationship between emissions of (a) ethanol and (b) ethyl acetate, and sugar concentration. *X* axis—percent sugar in a single fruit. *Y* axes—amount of chemicals in fruit scent emitted in 30 min, estimated based on calibration curves (see methods)

We first tested whether these two dominant compounds are correlated with sugar content. Ethanol had a weak, yet positive correlation with sugar content (linear regression: *F* = 5.33, *p* = .026, *r* ^2^ = .13, Figure 2a), while ethyl acetate had a negative correlation with sugar content (*F* = 9.72, *p* < .01, *r* ^2^ = .21, Figure 2b). This result is maintained when comparing total ester emissions and sugar level (not shown).

To further explore which VOCs characterize the sugar‐rich marula fruits, we ran a multivariate analysis based on the relative amounts of each compound within each sample. We found no statistically significant differences between trials with significant behavioral selections versus trials with nonsignificant behavioral selections (PERMANOVA: pseudo*F*_1,18_ = 1.49, *p* = .23).

Finally, to examine which scent compounds elephants may be using to select fruits, we conducted a SIMPER analysis to examine which compounds contribute to differences between selected and rejected marula fruits. Ethanol and ethyl acetate together constituted 74% of the total variance (Table 1), suggesting that these two compounds, which are correlated with sugar content, play a strong role in fruit selection.

**TABLE 1:** SIMPER analysis testing the contribution of each scent compound to fruit selection

Compound	Mean rejected	Mean selected	Av.Sq.Dist	Sq.Dist/*SD*	Contrib%	Cum.%
Ethanol	3.27E−02	−4.17E−03	5.76E−02	0.63	55.63	55.63
Ethyl acetate	8.50E−03	−9.23E−02	1.92E−02	0.93	18.56	74.2
Unknown sesquiterpene	−4.79E−05	3.67E−02	5.67E−03	0.5	5.48	79.68
Unknown alcohol	−1.13E−02	−2.96E−02	3.97E−03	0.59	3.83	83.51
Butanoic acid, 3‐methyl‐, ethyl ester	−1.69E−02	1.05E−02	3.52E−03	0.84	3.4	86.91
Butanoic acid, phenylmethyl ester	−6.89E−03	2.27E−03	3.09E−03	0.52	2.99	89.9
Cyclosativene	−1.93E−05	2.29E−02	2.07E−03	0.52	2	91.9

## 3 DISCUSSION

We conducted behavioral and chemical assays to test whether (a) elephants are capable of assessing fruit sugar levels based on scent alone; and (b) if they do so based on the amount of ethanol and aliphatic esters, which dominate marula odor profiles and correlate with sugar levels. The results of our behavioral experiments demonstrate that elephants are capable of detecting sugar levels in fruits based solely on scent. This demonstrates unprecedented olfactory performance in elephants: not only can they distinguish between plant species (Schmitt et al., 2018, 2020) and assess quantity (Plotnik et al., 2019), but they can also identify nuanced differences in the chemical composition of fruit scent within species, and ripeness category to infer small differences in sugar content. Elephants consistently chose fruits with higher sugar levels as long as the difference in the sugar content was larger than ~7%. It could well be that the absence of preference under this threshold is the result of indifference when the expected rewards are similar in quality, rather than an inability to identify sugar level. This is in contrast to other frugivorous species like primates, where an ability to discriminate between ripe and unripe fruits has been demonstrated (Nevo et al., 2015), but not an ability to infer sugar levels among fruits at a similar level of ripeness.

**FIGURE 3:**
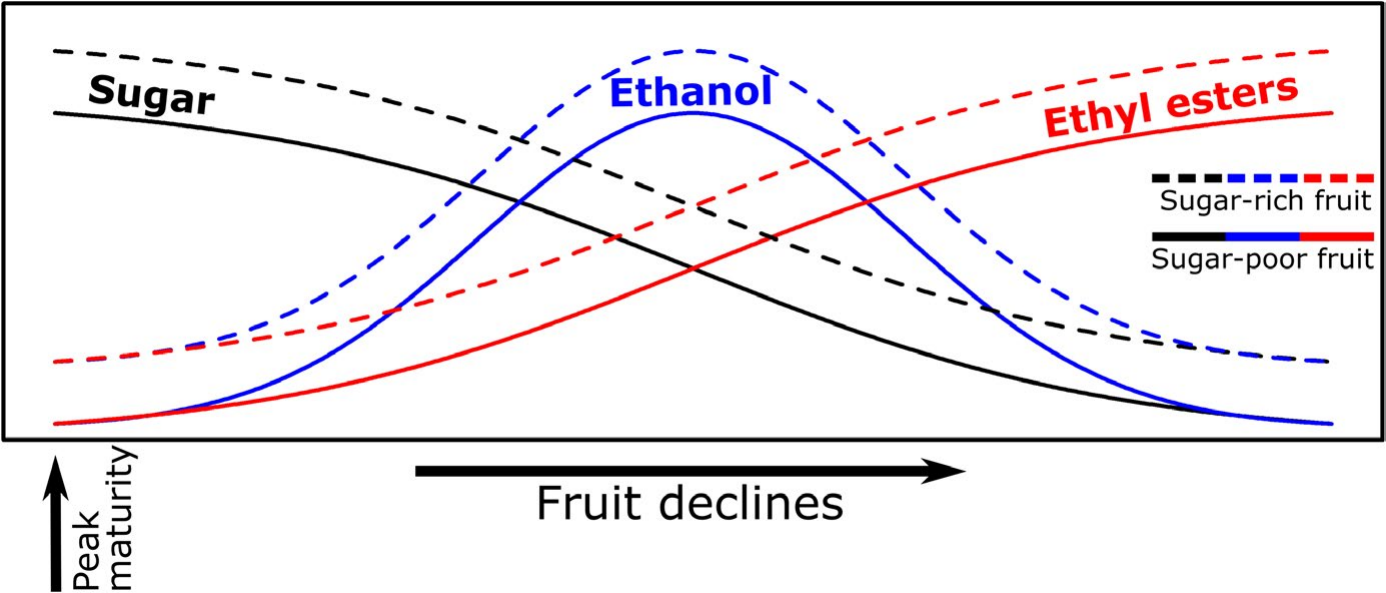
Theoretical model for the relationship between sugar, ethanol, and esters across marula maturation. The model begins at “peak maturity”—a time when the seed has matured and ready to be dispersed. Starting this point, anaerobic respiration by microbes would slowly reduce sugar levels and increase ethanol emission. Then, ethanol is converted to ethyl esters. Dashed lines represent a fruit whose starting point in terms of sugar levels was higher. Thus, higher amounts of esters are indicative of sugar levels, but only once accounting for fruit maturity, which would require another source of information to gauge, in this case ethanol

Our results also indicate that the ability to infer sugar levels in marula fruits is likely based on the perception of two main compounds: ethanol and ethyl acetate. Sugar levels were positively correlated with ethanol, but negatively with ethyl acetate. Ethanol is a product of anaerobic respiration by microorganisms inhabiting fruit, and increased sugar has been suggested to translate into higher ethanol levels (Dominy, 2004). Further, in line with our findings, elephants have been suggested to be attracted to ethanol and particularly rotting marula fruits (Janiak, Pinto, Duytschaever, Carrigan, & Melin, 2020). A more puzzling result is the negative relationship between sugar levels and ethyl acetate. Ethyl acetate is a direct biochemical product of ethanol through the alcohol acyltransferase pathway (Beekwilder et al., 2004), and we expected a positive relationship between them, not a negative one. Yet in line with our results, in cultivated oranges, it was shown that oranges infested with yeast contain less sugar and their scent is richer in esters (Peris, Rodríguez, Peña, & Fedriani, 2017). A possible scenario in this case is that after maturation, sugars begin to be slowly converted to ethanol, leading to an increase in ethanol and a concomitant decrease in sugar. Then, the ethanol is used as a substrate for ethyl esters (among them ethyl acetate), and the decline in sugar content is associated with a decline in ethanol levels while emission of ethyl esters peaks (Figure 3). In this scenario, higher levels of ester emission would indicate sugar content between different fruits if maturity level is held constant: in two fruits that matured around the same time, emissions of ethyl esters would be higher in the more sugary fruit, while within fruit, peak ester emission indicates that the fruit is already past its peak sugar concentration (Figure 3). Under this model, because of the gradual decline in sugar content, the amounts of esters on their own are not sufficient to determine fruit quality, and animals would need to rely on both ethanol and esters to identify sugar content. While our data does not permit us to test this, it can be tested by monitoring the emission of these compounds throughout the maturation process of fruits. At the same time, this simple model refers to the two dominant chemicals found in marula fruits. In both marula and other species, more nuanced information may be drawn from the concentration of other chemicals and their combination in a bouquet. Finally, this model may not be valid for all fruits, as the relationship between sugars, ethanol, and ethyl esters may be driven by other factors such as the fruit's susceptibility to microbial infection, its species‐specific biosynthetic machinery and its expression patterns.

In summary, our results demonstrate for the first time that a fruit‐eating animal can assess sugar levels based on scent alone. In our model system, this ability was apparent starting a relatively minor difference of 7% in sugar levels between fruits, and it remains for future studies to determine whether its absence below this threshold results from the absence of ability or motivation. We provide yet another demonstration of the keen sense of smell of elephants in a realistic food selection task. We speculate that elephants use two main compounds to assess sugar levels: ethyl acetate and ethanol. Our results also suggest that chemical fruit–frugivore communication is more complex than previously thought and that deciphering of fruit signals depends on estimation of the concentrations of multiple chemical compounds.

## 4 MATERIALS AND METHODS

### 4.1 Model system

We completed the behavioral experiments during March 2019 at the Adventures with Elephants facility near Bela Bela, Limpopo Province, South Africa. We used five semitame, yet wild foraging, subadult individuals ranging between 15 and 20 years old (three females, two males). Professional elephant handlers were used to ensure the comfort and safety of the elephants for all experiments.

We used marula fruits (*Sclerocarya birrea*: Anacardiaceae). Marula fruits are large and strongly protected fruits whose seeds are dispersed by African elephants (Midgley, Gallaher, & Kruger, 2012). Because our goal was to determine whether elephants are capable of recognizing nuanced differences in sugar content rather than simply discriminating between ripe, unripe, and over‐ripe fruits, we focused on fruits of a similar ripeness level. To determine which ripeness category of marula fruits (i.e., green, yellow, or brown) the elephants preferred to feed on, we conducted a set of experiments in which we placed a single fruit from differing ripeness categories (green, yellow, brown) in each of the two buckets and presented them to the elephants. This resulted in 3 different combinations (i.e., green vs. yellow, green vs. brown, and yellow vs. brown). Each elephant was allowed to make 5 selections for each combination tested. Our fruit ripeness experiment revealed that elephants strongly preferred yellow fruits over either green or brown fruits with yellow fruits being selected 88% and 96% of the time, respectively. We therefore used only yellow fruits in the subsequent experiments.

Fruits used in the experimental trials were collected on the day that they fell from the marula trees (i.e., when they were still green) and were allowed to ripen until yellow (~6 days). We collected 40 such fruits and randomly assigned them to 20 pairs that were used for twenty binary‐choice tests conducted with all five elephants (Tables S1 and S2). The sugar content of each fruit was unknown at the time of the experiment, but was later tested by an analytical laboratory. Odor samples were taken from each fruit (see below), which allowed us to compare potential odor cues with actual sugar content for each fruit used in a trial pair.

### 4.2 Choice experiment

We aimed to determine whether, within the yellow ripeness category, elephants selected for marula fruits that have higher sugar content based on odor cues alone. To do this, we used an olfactory‐choice experiment following the design of Schmitt et al. (2018) (Supplementary Video S1).Our experiment used two identical ~120 L plastic bins placed side by side that each had a PVC lid with 200 holes (1 cm diameter) drilled into them. This prevented the elephants from touching or seeing what was inside each bin, thus ensuring that the elephants could only use olfactory cues to make their selections. Inside each bin, we placed a single marula fruit in the center.

To guarantee that the elephants could not see the experimental set‐up during the trials, a professional handler instructed the elephants to face away (180 degrees) from where the bins were lined up. Once fruits were placed inside each bin, the bins were arranged side by side. As per Schmitt et al. (2018), the elephant being tested was then instructed to turn, face forwards and to “smell” the bins. At this point, the elephant would step up to the bins and place its trunk on each PVC lid and inhale the odors from each fruit held within. After smelling both bins, the elephant was instructed to remove its trunk. The elephants were then instructed “choose.” To indicate their selection, the elephants placed their trunk on the bin containing the fruit that they preferred. All elephants were familiar with the experimental procedure (scent‐based choice between two items, in which they receive only the item they indicate after sniffing the two) and have gone through multiple similar choice tests over the years, although never with marula fruits.

To reinforce the choice, we gave the elephant a fruit that was at a similar ripeness stage to the fruit inside the selected bin. We repeated this procedure five times consecutively for every elephant for each combination (we accounted for serial correlation in our statistical analyses, see below). To ensure hunger levels did not influence the selection between fruits, the elephants were allowed to forage naturally for 1 hr prior to testing. We used a random number generator to randomize the position of each fruit, and the handler adjacent to each bin (who also did not know which fruit was in their bins). The experimenter was also blind to the position of each fruit. All experiments with a single pair of fruits were conducted consequently, and all elephants were tested on a single pair within 1 hr, thus ensuring that the fruits did not go through significant maturation and all elephants responded to comparable stimuli. Bin lids were wiped between each run to ensure that elephants do not cue on their own saliva, and the bins were cleaned with water and a clean towel between trials with different fruits.

All aspects of this research were approved by the Duke University Institutional Animal Care and Use Committee (IACUC) (Reference number: A248‐18‐10).

### 4.3 Scent sampling and fruit chemistry

Following the behavioral tests, we sampled the scent of the fruits and then the sugar content of their pulp.

Fruit scent was sampled using semidynamic headspace technique as described in Nevo et al. (2018). Individual fruits were placed in a chamber made of an unused 40 cm oven bag (Toppits, Germany) which was sealed on one side with a zip‐tie. On the other side, it was sealed around a teflon tube on which we mounted a self‐produced volatile‐organic compound (VOC) trap. The traps were made of a Quartz tube (30 mm long, 3 mm in diameter) that included 1.5 mg Tenax TA 60–80 mesh, 1.5 mg Carbotrap B 20–40 mesh, and 1.5 mg Carbosieve S‐III 60–80 mesh (all Sigma Aldrich), locked between layers of glass wool. Fruits were incubated in the chamber for 20 min in room temperature. Then, the air containing all VOCs was pulled through the VOC trap using a self‐constructed vacuum pump connected to the teflon tube for 10 min at 200 ml/min. Traps were then placed in 1.5 ml glass vials sealed with teflon caps and kept frozen until analyzed, with the exception of transportation in isolation with ice packs for about 15 hr.

Samples were analyzed on an Agilent 7890B gas chromatograph (GC) equipped with an Agilent DB‐WAX polar capillary column (30 m long; 0.25 mm in diameter; film thickness 0.25 µm), coupled with an Agilent 5977A mass spectrometer (MS) operating at electron ionization mode. Samples were introduced into the thermal desorption unit (Gerstel) at 30°C. After 1 min, it started to heat at 100°C/ min until it reached 310°C, a temperature on which it rested for 8 min. TDU transfer line temperature was set to 320°C. Released VOCs were then transferred to a cold injection system (Gerstel) in which the liner was cooled to −100°C using liquid nitrogen. The liner began heating at 12°C/min until it reached 250°C, a temperature on which it rested for 8 min. Samples were introduced to the column using a 1:20 split (i.e., 95% of the sample was sent to the column) using the solvent vent mode (Gerstel). Initial oven temperature was set to 30°C. After a 1 min hold time, it began heating at 10°C/ min until it reached 240°C and then rested on this temperature for 30 min. MS source temperature was set to 230°C and MS quad to 150°C. These settings were determined following an optimization process.

Sample outputs were analyzed using Amdis 2.71. Compound were identified using their retention index and mass spectra, using the NIST 11 library. We excluded all VOCs that were found in similar amounts in control samples (empty oven bags, sampled in identical conditions), as well as known contaminants like phthalates and siloxanes. The amounts of the compounds were determined by calculating their peak area. For two compounds (ethanol, ethyl acetate), we also calculated absolute amounts by creating a calibration curve based on a ladder of concentrations of synthetic standards.

Following odor sampling, fruits were frozen for sugar content analysis. Sugar content was determined using an enzymatic method (Marais, De Wit, & Quicke, 1966) at the Cedara Feed Analysis Laboratories.

### 4.4 Statistical analyses

#### 4.4.1 Behavioral analyses in relation to sugar content

All of our behavioral choice experiments included a series of binary choices (i.e., selecting for fruit A vs. fruit B). As a result of using the same 5 elephants for all trials, we treated each individual as the subject for repeated measures in generalized estimating equations (GEEs). We used GEEs to account for potential nonindependence of our data, which could stem from an individual possibly remembering previous trials. GEEs use a population‐level approach based on a quasilikelihood function and deliver population‐averaged estimates of the parameters. The coefficients of GEE regressions are marginal effects (i.e., the average effects across all the subjects in the data; see Wang 2014). For our study, the GEEs model the proportion of elephants that make a given choice and compare this to an expected 50% distribution expected under random selection for a given choice. The model incorporated an exchangeable correlation matrix and binomial error distribution with a logit link function. We then back‐transformed data from the logit scale for graphical representation, which results in asymmetrical confidence intervals (CIs; Hardin, 2005).

To specifically determine whether elephants could make diet selections based on sugar content within the presented fruits, we analyzed the proportion of elephants that chose the option with higher sugar content (as determined *post hoc* via wet chemistry). We used means and their 95% CIs to establish whether the elephants' preference between the fruits differed from the expected 50% distribution under random selection for each fruit available. Specifically, we used GEEs to answer 2 key questions: (a) whether elephants show preference for items with proportional difference in percent sugar content and (b) whether elephants show preference for items with greater rank difference in percent sugar content. These two offer two separate perspectives toward the question whether elephants can identify sugar levels based on scent alone. The first explores relative differences and is fully parametric, while the other focuses on differences in absolute amounts. In the latter, our approach required relying on differences in ranks, that is, a nonparametric variable. A consistency with the percentage differences would therefore indicate that the results are not driven by outliers. We used separate GEEs to test the above questions. We used rank difference in percent sugar content and proportional difference in percent sugar content as independent variables with binomial choice as the response variable. We ran a separate analysis on fruit mass to ensure that elephant selection was not influenced by the size of the fruit and determined that this was nonsignificant. GEE models were run on SPSS V25.

#### 4.4.2 Sugar and scent chemistry, behavioral analyses in relation to scent

To link sugar content and potential compounds emitted from fruits that allow elephants to make informed diet choices, we first tested the relationship between sugar and the two dominant components of marula scent: ethanol and ethyl acetate. We used regression models on all 38 individual fruits, where percent sugar was the independent variable, and the amounts of ethanol or ethyl acetate were the dependent variable. We ran a similar model to determine if the effect of the total amounts of esters differs from the effect of ethyl acetate alone, but found identical results because of the strong dominance of this one ester in marula scent.

To further explore whether any other compounds in marula scent predict sugar levels, we ran a multivariate analysis based on the relative amounts of each compound within each sample. Prior to running any of the multivariate models, we first created a dataframe that reflected the difference in odor profile for each pair of fruits (i.e., a single column of data reflecting the difference in a particular compound for each pair). To create this dataframe, we subtracted the percent contribution value for each VOC found in the fruit with lower sugar content from the value from the fruit with the higher sugar content within a pair. This yielded a single table that reflected the difference in percent contribution of a given VOC across all 19 pairs of fruits (one odor sample was lost when the glass probe broke, thus leaving us with 19 trials instead of 20). Negative values reflect VOCs that were higher in the fruit with lower sugar content. Upon exploring the data, we found that Caryophyllene appears to be a random and uncommon compound that happens to be in only 3/40 fruits, 2 in a selected fruit and 1 in a nonselected fruit. Because it happens to be in a high concentration in 2 of the preferred fruits, it skews the data significantly. As a result, we removed it from our analyses to reflect more consistent VOC patterns. These values were then used in the multivariate analyses. We also included an additional column that noted whether there was a significant selection for a fruit for each pair.

We used a PERMANOVA (using Euclidean distances because we had both positive and negative values) to examine differences in the odor profiles for trials that had significant selection versus trials that did not. Although we found no significant differences between groups (See Results), the behavioral choice experiment suggests that there are differences between the odor profile of the groups. Thus, we explored the role that individual VOCs play in characterizing the odor profiles of trials that had significant versus nonsignificant behavioral selections using SIMPER analysis. We used the similarity percentages (SIMPER) function in Primer (Clarke & Gorley, 2006), which identifies compounds that contribute most to the mean similarity within a particular group and mean dissimilarity between groups (i.e., trials with significant behavioral selections versus trials with nonsignificant behavioral selections). SIMPER analysis was ran on Primer, and PERMANOVA on R (code attached).

## CONFLICT OF INTEREST

The authors declare no competing interests.

## AUTHOR CONTRIBUTIONS

**Omer Nevo:** Conceptualization (equal); data curation (equal); funding acquisition (lead); investigation (equal); methodology (equal); project administration (equal); resources (equal); software (equal); writing – original draft (equal); writing – review & editing (equal). **Melissa H. Schmitt:** Conceptualization (equal); data curation (equal); formal analysis (equal); funding acquisition (equal); investigation (equal); methodology (equal); project administration (equal); software (equal); visualization (equal); writing – original draft (equal); writing – review & editing (equal). **Manfred Ayasse:** Methodology (equal); validation (equal); writing – review & editing (equal). **Kim Valenta:** Conceptualization (equal); project administration (equal); writing – original draft (equal); writing – review & editing (equal).

## Supporting information

Table S1‐S2

Video S1

Supplementary Material

## Data Availability

All raw data used for the analyses are available in Tables S1 and S2.
