# Supplementary material for: Sweet tooth: Elephants detect fruit sugar levels based on scent alone
Source: Ecol Evol. 2020 Sep 11;10(20):11399–407. doi: 10.1002/ece3.6777 (PMC7593167; doi:10.1002/ece3.6777)
Supplement: Supplementary file 3 — Supplementary Material [file ECE3-10-11399-s003.docx]

Marula PERMANOVA analysis

MH Schmitt and O Nevo

August 6, 2020

# PERMANOVA analysis

The following analysis was used to analyze whether trials where African elephants (*Loxodonta* *africana*) showed significant preference for a given marula fruit differed in their VOC compounds. This analysis accompanies Nevo et al. 2020. Sweet tooth: elephants detect fruit sugar levels based on scent alone. Ecology and Evolution.

#### Load library

library(vegan)

## Loading required package: permute

## Loading required package: lattice

## This is vegan 2.5-4

library(plyr)
library(reshape2)

#### Load data

The below dataframes can be found in Supplementary Materials under the same names.

compounds<-read.csv("Marula all compounds dataframe.csv")
compound.var<-read.csv("all compounds factor.csv")

#### Create matrix

matrix.compounds = compounds

#### Modify matrices

row.names(matrix.compounds) <-compounds$X
matrix.compounds <- matrix.compounds[ -c(1) ]
euclid = vegdist(matrix.compounds, "euclid")

#### Grouping terms

names(compound.var)

## [1] "selected" "trial"

Species <-compound.var$trial
Species <-as.factor(Species)
Preference<-compound.var$selected
Preference<-as.factor(Preference)

#### Permanova

permanova = adonis2(euclid ~ Preference)
permanova

## Permutation test for adonis under reduced model
## Terms added sequentially (first to last)
## Permutation: free
## Number of permutations: 999
##
## adonis2(formula = euclid ~ Preference)
## Df SumOfSqs R2 F Pr(>F)
## Preference 1 7.2379e+16 0.08066 1.4915 0.226
## Residual 17 8.2498e+17 0.91934
## Total 18 8.9735e+17 1.00000

##### END
